# Neuroprotective Potential of Ceftriaxone Alone and in Combination with Minocycline in Sporadic Alzheimer's Disease

**DOI:** 10.1002/alz70855_102687

**Published:** 2025-12-23

**Authors:** Shilpa Kumari, Rahul Renukadas Deshmukh

**Affiliations:** ^1^ Central University of Punjab, Bathinda, punjab, India; ^2^ Maharaja Ranjit Singh Punjab Technical University, Bathinda, Punjab, India; ^3^ Department of Pharmacology, Central University of Punjab, Bathinda, Bathinda, Punjab, India

## Abstract

**Background:**

Alzheimer's disease is a progressive neurodegenerative disorder characterized by a decline in cognitive and memory functions. The extracellular deposits of β‐amyloid and intracellular accumulation of hyperphosphorylated microtubular tau protein are the two major hallmarks of AD. There is a progressive deterioration of memory, cognition, and behavior that affects the daily activities and life of AD patients. Various pathogenetic mechanisms have been implicated in AD progression such as Aβ plaque accumulation at the synapse, tau tangle formation inside neurons, increased glutamate concentrations at synapse causing excitotoxicity, oxidative stress, neurotransmitters deficits, and neuroinflammation, etc.

**Method:**

Here in the present study, we have explored the neuroprotective potential of ceftriaxone alone and in combination with minocycline, against the intracerebroventricular streptozotocin (ICV‐STZ) induced experimental sporadic Alzheimer's disease. Bilateral ICV‐STZ (3 mg/kg) infusion in rats produces AD‐like symptoms. The spatial and non‐spatial memory was evaluated using the Morris water maze (MWM) and object recognition test (ORT). The ceftriaxone (200 mg/kg/i.p) was administered alone and in combination with minocycline (50 mg/kg/i.p) daily for 7 days from day 14^th^ ‐ day 28^th^ after ICV‐STZ administration.

**Result:**

The ICV‐STZ administration produced cognitive and memory deficits as indicated by a significant elevation in markers of oxidative stress and degenerative changes in the hippocampus and cortex regions of the brain as shown by biochemical and neurochemical estimation. However, a combination of ceftriaxone (200 mg/kg/i.p) with minocycline (50 mg/ kg/i.p) treatment significantly attenuates the STZ induced decline in memory, oxidative stress and thus prevents hippocampal and cortical neuronal loss.

**Conclusion:**

The observed outcomes of the present study suggest the synergized neuroprotective potential of the ceftriaxone with minocycline in cognitive and memory dysfunctions.

